# Trapping tiny pollutants: SERS-driven strategies for microplastics and nanoplastics detection

**DOI:** 10.1016/j.isci.2025.113888

**Published:** 2025-10-28

**Authors:** Jayasree Kumar, Phularida Amulraj, Sadia Fatima Haroon, Rangabhashiyam Selvasembian, Venugopal Rao Soma, Rajapandiyan Panneerselvam

**Affiliations:** 1 Raman Research Laboratory (RARE Lab), Department of Chemistry, SRM University-AP, Andhra Pradesh, Amaravati 522240, India; 2 Department of Environmental Science and Engineering, School of Engineering and Sciences, SRM University-AP, Amaravati, Andhra Pradesh 522240, India; 3 DRDO Industry Academia – Centre of Excellence (DIA-CoE, Formerly ACRHEM), University of Hyderabad, Hyderabad 500046, Telangana, India; 4 School of Physics, University of Hyderabad, Hyderabad 500046, India

**Keywords:** analytical chemistry instrumentation, environmental monitoring, pollution

## Abstract

Microplastics and nanoplastics are almost everywhere in biological and environmental systems, posing serious risks to human health and ecology. However, due to their complex matrices, varied sizes, and morphologies, their detection and quantification remain challenging. Particularly, Raman and surface-enhanced Raman spectroscopy (SERS) hold great promise for the detection, characterization, and quantification of micro/nanoplastics. In this review, we introduce the Raman and SERS fundamental principles, instrumentation, and SERS substrate design strategies. Particularly, emphasis is placed on SERS-enabled ultrasensitive detection, integration with chemometrics and machine learning tools, culminating in the real-world applicability. Additionally, we elaborate on the current limitations, including signal variability, lack of standardization, and sample preparation challenges. Finally, future directions involving artificial intelligence (AI) integration, substrate engineering, and multi-modal analytical approaches are discussed.

## Graphical abstract

**Figure:**
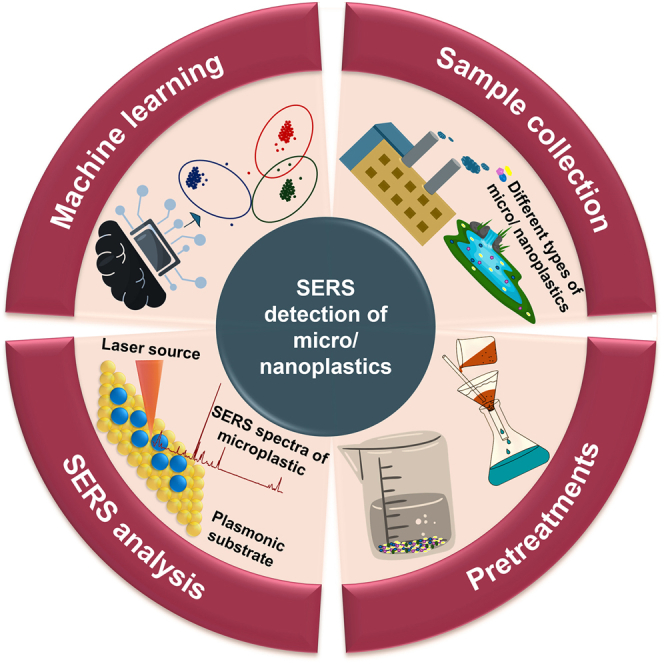

## Introduction

Microplastic (MP) pollution has drawn considerable scientific attention as a serious and widespread environmental issue.^1^^,^^2^ In 1972, Carpenter et al. visually identified marine plastic debris larger than 300 μm along the coastal regions from western Long Island Sound to Vineyard Sound, documenting the early presence of plastic pollutants in the marine environment.^3^ Decades later, in 2004, Thompson et al. coined the term “microplastics” and conducted one of the first comprehensive studies on microscopic plastic fragments in environmental samples.^4^ They employed Fourier Transform Infrared (FT-IR) spectroscopy to characterize and identify common plastic polymers, including acrylic, alkyd, poly(ethylene: propylene), polyamide (nylon), and polyester. This pioneering work not only confirmed the widespread presence of microscopic plastic particles but also catalyzed global research interest in the detection and environmental impact of microplastics.^4^ In recent years, numerous investigations have revealed the extensive presence of plastics in a variety of matrices, including drinking water,^5^ lake water,^6^ seawater,^7^ beach sand,^7^ and food products.^8^ Plastic pollution comprises a diverse spectrum of particle sizes categorized as macroplastics (≥2 cm), mesoplastics (5 mm - 2 cm), MPs (1 μm - 5 mm), and nanoplastics (NPs) (<1 μm). Given their potential for bioaccumulation, environmental persistence, and related toxicological effects, micro- and NPs are especially alarming,^9^ including oxidative stress, inflammation, disruption of cellular and endocrine functions, and potential neurotoxicity.^10^^,^^11^ MPs possess high surface area-to-volume ratios and colloidal properties,^12^ facilitating the adsorption of organic pollutants,^13^ microbial pathogens,^14^ and toxic metal ions.^15^ These particles can infiltrate the human body via ingestion, inhalation, or water consumption, with evidence of their presence in biological matrices such as breast milk and the placenta, raising substantial health concerns.^16^^,^^17^^,^^18^

**Figure 1:**
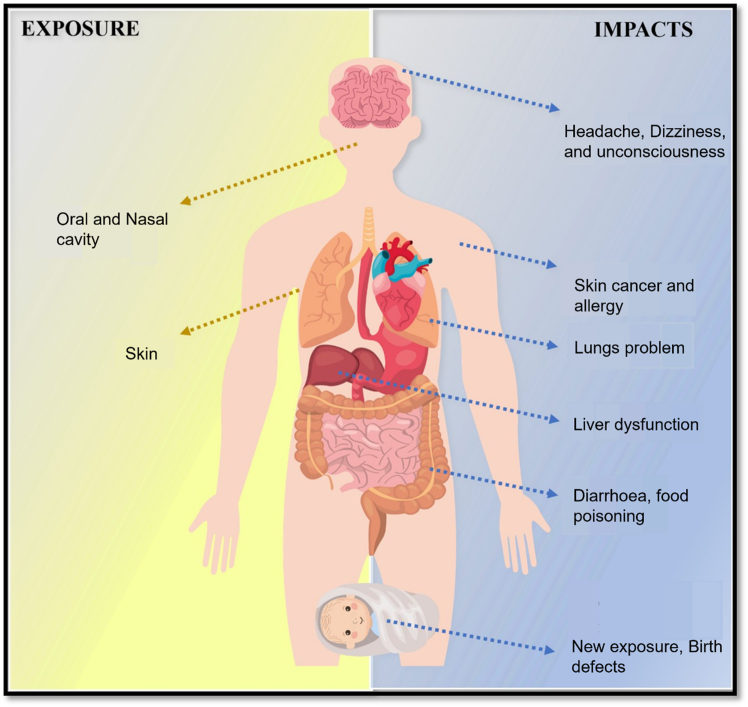
Schematic illustration of MP exposure routes and their associated physiological effects on the human body

A comprehensive evaluation of plastic pollution and its fate over the past two decades was excellently addressed in the review by Thompson and his group.^19^ Figure 1 illustrates the physiological effects of MP exposure and its associated implications for human health. With the escalating global concern over plastic pollution, researchers have devoted significant efforts to developing effective methods for detecting MPs in environmental samples. One of the key challenges faced by the scientific community is the reliable identification and quantification of MPs in complex and heterogeneous matrices. Several years have been spent developing advanced and reliable instrumentation techniques capable of both detecting and quantifying microplastics.^20^^,^^21^^,^^22^ Initial studies primarily focused on the chemical and microscopic techniques within controlled laboratory settings, which are not suitable for real-world sample analysis due to matrix complexity and sample variability. This has led to a growing emphasis on developing reliable and standardized strategies for assessing, detecting, and analyzing microplastics in complex environmental and real-world samples.^23^

In this context, surface-enhanced Raman spectroscopy (SERS) has garnered growing attention—offering exceptional signal enhancement (often up to factors of 10^8^), high molecular specificity, rapid detection capability, and promising miniaturization for field deployment—making it particularly well-suited for trace-level MPs/NPs detection in challenging matrices.^24^ In the recent years, there has been a significant research impetus focused on developing analytical methods for the detection and characterization of plastics in various matrices.^25^

For instance, Fourier transform infrared spectroscopy (FTIR),^26^ Raman spectroscopy (RS),^27^ SERS,^28^ coherent anti-Stokes Raman spectroscopy (CARS),^29^ near-infrared spectroscopy (NIR)^30^ laser-induced breakdown spectroscopy (LIBS),^31^ Quartz-enhanced photoacoustic spectroscopy (QEPAS),^32^ hyperspectral imaging (HSI),^33^ optical microscopy,^34^ scanning electron microscopy (SEM),^35^ transmission electron microscopy (TEM),^36^ thermogravimetric analysis (TGA),^37^ differential scanning calorimetry (DSC),^38^ pyrolysis-gas chromatography-mass spectrometry (Py-GC-MS),^39^ thermal extraction desorption-GC-MS (TED-GC-MS),^40^ evolved gas analysis (EGA),^41^ gas chromatography-mass spectrometry (GC-MS),^42^ high-performance liquid chromatography (HPLC),^43^ size exclusion chromatography (SEC),^44^ liquid chromatography-mass spectrometry (LC-MS),^39^ staining (e.g., Nile Red),^45^ and flow cytometry were used to detect micro/nano plastics.^46^ Among various analytical techniques, RS^47^^,^^48^ SERS enjoys the reputation of a powerful analytical technique for the identification of various polymer types, including polypropylene (PP), polyethylene (PE), polyethylene terephthalate (PET), polystyrene (PS), and others.^49^^,^^50^ The integration of RS with microscopic imaging (micro-Raman) enables simultaneous identification, quantification, and spatial visualization of MP particles.^51^ Despite these advantages, RS faces limitations in the detection of extremely small micro- and NPs due to weak scattering signals, leading to lower sensitivity and higher detection limits.^52^

**Figure 2:**
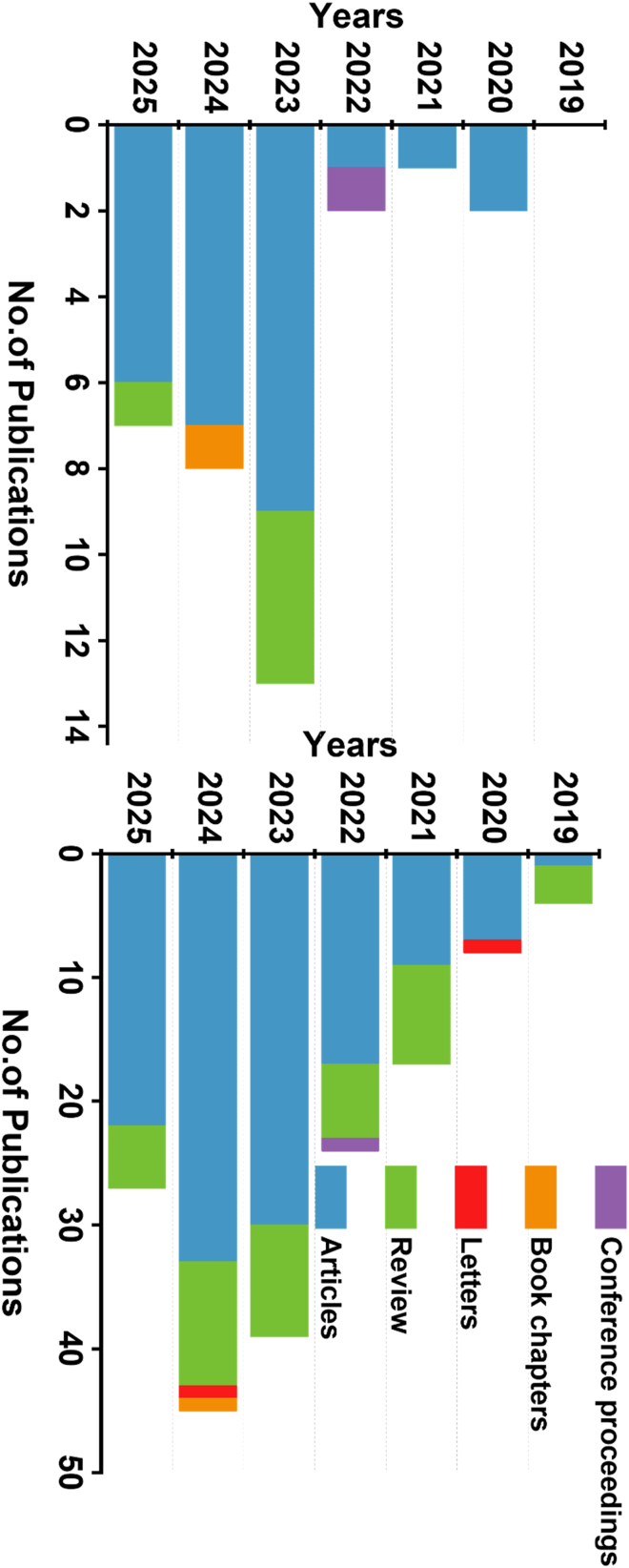
The number of publications retrieved from SCOPUS using the keywords “Raman, microplastic, nanoplastic detection, and SERS, microplastic detection” from 2019 to 2025

Significantly, SERS^53^ has become an efficient technique to overcome the drawbacks of traditional RS, offering improved sensitivity, portability, efficiency, robustness, and ease of use.^6^^,^^54^^,^^55^ SERS offers unparalleled sensitivity and specificity, allowing the detection and identification of micro- and NPs at trace levels.^56^^,^^57^ By utilizing molecular vibrational fingerprints, SERS provides a non-invasive, molecule-specific platform capable of differentiating various types of MPs, a task that remains challenging for most other techniques.^58^^,^^59^^,^^60^^,^^61^ Additionally, the non-interfering nature of water in SERS analysis makes it particularly well-suited for aqueous samples.^62^^,^^63^^,^^64^^,^^65^ As illustrated in Figure 2, there has been a significant increase over the past decade in the number of research articles, review articles, letters, book chapters, and conference proceedings focusing on the application of Raman and SERS for MP detection.

In this review, we begin by presenting the various detection techniques employed for the analysis of micro- and nanoplastics. This is followed by a discussion on the advantages of SERS, along with the fundamental principles of Raman and SERS techniques. The main intent of the review is to discuss how these techniques can be equipped for the sensitive and specific identification of micro/nanoplastics. We further discuss the applications of colloidal and planar SERS substrates for detecting plastics at ultralow concentrations. Subsequently, we highlight applications involving real-world sample analysis and provide a critical evaluation of current challenges in the field. Finally, we propose potential strategies to address these limitations and emphasize the need for interdisciplinary collaboration for practical applications.

### Detection methods

**Figure 3:**
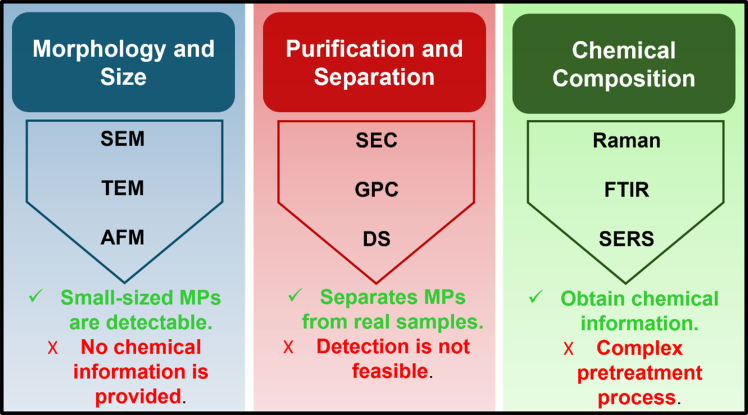
Schematic representation of various strategies employed for the detection and analysis of MPs

Several researchers have focused on developing novel and sensitive detection strategies for the accurate identification and quantification of MPs.^66^ In this context, analytical approaches employed in MP research can be broadly categorized into three major groups: (i) determination of morphology and size using imaging techniques, (ii) purification and separation processes, and (iii) analysis of chemical composition using spectroscopic techniques. An overview of these strategies, along with the corresponding analytical techniques, is presented in the schematic illustration (Figure 3) and summarized^67^^,^^68^^,^^69^^,^^70^^,^^71^^,^^72^^,^^73^ in Table 1.

**Table 1:** Summary of various analytical techniques employed for the detection of MPs, highlighting their advantages and limitations

Detection technique	Nature of the micro-/NPs	Advantages	Limitations
SEM^35^	PE, PET, PS, PU, PC, PP, PAN, PA, PVC	High-resolution and elemental analysis are possible with EDS	Expensive and tedious sample preparation
STXM and TEM^67^	PET	Very high resolution (<0.1 nm)	Requires sample preparation for particle size >100 nm
AFM^36^	PET, PVC, PS	Preserved sample surface	Quantification is not possible
DS^68^	PA, PVC, PET	Inexpensive	Detection not possible
SEC^44^	PS	High-quality separation	Detection not possible
GPC^69^	PS	High-quality separation	Difficulty in taking separate samples
Py-MS^70^	PS, PP	Detection of additives and degradation products is possible	Sample destruction
ICP - MS^71^	PS	Sensitive detection of additives and degradation Products is possible	Sample destruction and complex sample analysis
ASAP - MS^72^	PA 6, PET, PS	Sensitive detection of Additives and Degradation Products is possible	Sample destruction and complex sample analysis
FTIR^73^	PET	Non-destructive and chemical confirmation of particles	Interference with water
RS^29^	PA, PE, PET, PS, PP, PVC	Non-destructive and chemical confirmation of particles	Filtration is needed
SERS^63^	PS, PE, PTFE, Nylon, PMMA, PET	High sensitivity	Non-uniform signals for different sizes

### Imaging techniques

To investigate the morphology and size of plastic particles, conventional optical microscopy techniques have been widely employed to obtain information on the shape, size, and color of plastic particles.^74^ However, they are generally ineffective for analyzing NPs and cannot reveal molecular or chemical composition.^75^^,^^76^ Advanced imaging techniques such as SEM^77^ and TEM^78^ are commonly employed for high-resolution morphological analysis at the nanoscale.^35^^,^^79^^,^^80^^,^^81^^,^^82^ SEM, when coupled with energy-dispersive X-ray spectroscopy (EDS), has been effectively utilized for the detection of NPs, enabling elemental mapping that assists in distinguishing carbon-rich plastic particles from the surrounding matrix.^83^ Additionally, the integration of SEM with RS offers a powerful analytical approach that combines high-resolution imaging with chemical characterization, allowing the comprehensive screening of NPs based on both their structural and molecular features.^84^ Other imaging techniques, such as the optical sieve, provide rapid and accessible visualization for micro- and nanoplastic sizing and counting but face limitations in chemical specificity, performance in complex matrices, and resolution below ∼100 nm.^85^

### Separation techniques

The separation of MPs from environmental matrices is a crucial preliminary step for their effective detection and identification. These separation techniques are typically employed during the sample preprocessing stage. For example, size exclusion SEC^44^ is commonly used to isolate micro- and NPs, often employing fluorescently labeled particles to facilitate tracking. Similarly, Density Separation (DS)^68^ and Gel Permeation Chromatography (GPC)^69^ are conventional approaches to segregating MPs based on size or density differences. However, these methods are generally limited to the separation step and do not provide detailed chemical identification of the particles. Therefore, separation techniques are often coupled with advanced analytical methods such as spectroscopy or mass spectrometry to enable comprehensive detection and characterization of microplastics.

### Spectroscopic techniques

Both IR^73^ and RS^29^ possess significant advantages for identifying MP particles by providing detailed chemical information through the characteristic vibrational fingerprint of polymeric materials.^86^^,^^87^^,^^88^ These vibrational spectroscopic techniques enable non-destructive analysis while offering molecular-level insights into the chemical and structural composition of MPs. However, conventional RS faces significant limitations in detecting plastic nanoparticles due to their extremely small size and inherently weak scattering signals. Recently, advancements including the development of microextraction methods for isolating MPs and hydrophobic pollutants from seawater, as well as the application of Raman tweezers capable of detecting particles as small as 50 nm, have notably enhanced the sensitivity and applicability of Raman-based detection.^46^^,^^89^ In this context, SERS has emerged as a robust technique for both qualitative and quantitative analysis of MPs, owing to its exceptional signal amplification capabilities.

Other notable methods include pyrolysis mass spectrometry (Py-MS),^39^ mass spectrometry (MS),^70^ thermogravimetric analysis coupled with Fourier transform infrared spectroscopy, and gas chromatography-mass spectrometry (TGA-FTIR-GC-MS).^90^ These approaches have significantly advanced the qualitative and quantitative measurement of MPs in environmental samples. However, these techniques suffer from certain limitations. For instance, Py-MS is suitable for small sample sizes (e.g., 0.5 mg) but remains impractical for large-scale environmental studies.^91^ MS often involves complex and labor-intensive sample preparation.^70^ Conventional techniques for microplastic detection face several limitations, including water interference and uniformity issues. FTIR typically shows higher limits of detection (LOD), e.g., 44 MP/L, with recovery rates around 91%, but is prone to interference from water and organic matter, which can affect spectral accuracy.^92^ Similarly, Py-GC-MS, while providing polymer-specific identification, suffers from matrix effects and sample preparation losses that can reduce recovery and lead to non-uniform detection, especially in complex environmental matrices.^93^ These limitations underscore the challenges of conventional methods and highlight why SERS, with proper substrate optimization, offers enhanced sensitivity, minimal water interference, and potential for lower LODs in trace microplastic detection.

SERS stands as a promising tool for detecting microplastics in many ways, offering several advantages over the aforementioned methods.^94^ The major advantage of SERS lies in its high sensitivity and specificity, which provide a way to detect different types of polymers at trace-level concentrations, overcoming the limitations of most techniques discussed earlier.^95^ Additionally, SERS offers the potential for quantitative detection and no water interference with appreciable uniformity and reliability of samples,^96^ which sets it apart from other analytical techniques.^97^ This enables the screening of environmental samples without extensive laboratory pre-treatments. The integration of portable Raman instruments for real-time field analysis has the potential to revolutionize the detection and monitoring of microplastics in real samples, providing immediate insights into contamination levels across various matrices.^24^^,^^96^^,^^98^^,^^99^

## Principles of Raman and surface-enhanced raman spectroscopy techniques

The Raman effect was reported experimentally in 1928 by Indian physicists C. V. Raman and K. S. Krishnan, who identified what they described as *“a new type of secondary radiation,”* now known as Raman scattering. This phenomenon arises when matter interacts with electromagnetic radiation, shifting the energy of the incident photon.^100^ RS is an analytical method based on inelastic light scattering at the molecular level. When incident light (frequency ω_0_) interacts with a molecule, it excites the vibrational energy levels, transitioning the molecule from its ground state to an unstable excited state.^101^ The molecule then relaxes to a lower energy state by emitting scattered photons (frequency ω_R_). The energy difference between ω_0_ and ω_R_ correlates with the vibrational energy of the molecule. This shift in frequency (ω_0_ ± ω_R_) is reflected in the Raman spectrum as the vibrational peak. As such, Raman scattering offers significant advantages in probing molecular vibrations and rotations. However, due to its inherently low scattering efficiency, Raman scattering demands a large sample volume and extended acquisition times for spectrum collection.^102^ One of the main advantages of RS is its ability to offer chemical and fingerprint information without interference from carbon dioxide and water. However, a major limitation is that Raman scattering is inherently weak, with probe molecules exhibiting relatively low scattering cross-sections. As a result, RS remained underutilized by researchers for over two decades following its initial discovery. The advent of lasers in 1960 significantly enhanced the sensitivity of RS, leading to a substantial improvement in its detection limits. The samples are illuminated with a laser light source, and the subsequent Raman scattering is detected by a spectrometer equipped with a photosensitive detector after passing through the target material.^103^ This breakthrough enabled the technique to be applied effectively for bulk sample analysis.^104^ RS, however, was unable to identify surface species or lower concentrations of analytes.

The first instance of SERS was reported by Fleischmann, Hendra, and McQuillan in 1974, who observed electrochemical potential-dependent Raman spectra from pyridine molecules adsorbed on a roughened silver electrode.^105^ This pioneering work stemmed from their efforts to apply RS to electrochemical systems.^105^ In 1977, Jeanmaire and Van Duyne^106^ independently proposed the electromagnetic mechanism for SERS. In the same year, Creighton and Albrecht^107^ introduced the chemical (or charge-transfer) mechanism involving surface resonance effects. Later, in 1978, Moskovits provided the foundational explanation of the influence of surface plasmons in metal nanostructures on the enhancement of SERS. He specifically examined roughened Ag electrodes and predicted that similar plasmonic effects could occur in Ag and Cu colloids interacting with adsorbates.^108^ This hypothesis was experimentally validated in 1979 by Creighton and colleagues, who demonstrated the plasmonic enhancement using Ag and Au colloids. Their work extended the scope of SERS research from roughened electrode surfaces to colloidal metal nanostructures, emphasizing the pivotal role of plasmonic nanostructures in SERS.^109^ Interestingly, the plasmonic properties of colloidal metal nanoparticles were first noted much earlier, in 1857, when Faraday observed reversible color changes in thin Au films prepared from colloidal Au nanoparticles under mechanical pressure, shifting from bluish-purple to green.^110^ Free-electron-like metals such as Au, Ag, and Cu act as nanoscale resonators in SERS, amplifying the target analyte molecules’ Raman signals. This amplification enables the detection of analytes at extremely low concentrations (nM, pM, fM, and Attomolar), going down all the way to the single-molecule level. The enhancement of Raman signals in SERS arises from two primary mechanisms: electromagnetic enhancement (EM),^111^^,^^112^ and chemical enhancement (CE).^113^^,^^114^^,^^115^^,^^116^

Among the mechanisms causing the SERS enhancement effect, EM enhancement is the most important, whereas the CE mechanism is rather minor. The plasmonic characteristics of metal nanostructures are responsible for the EM amplification.^117^ It operates as a long-range effect confined within the evanescent field (<5 nm), exhibiting an exponential decay with increasing distance between the adsorbed molecules and the SERS substrate. Through the EM effect, the Raman signals of the adsorbed molecules can be amplified by as much as 6 to 8 orders of magnitude. In contrast, the CE mechanism functions as a short-range phenomenon occurring at the Angstrom scale, involving direct interactions between the probe molecules and the SERS substrate. This enhancement arises from the transfer of charge between the analyte molecules and the plasmonic nanostructures, inducing a notable change in molecular polarizability. The CE mechanism typically enhances the Raman signal by up to 2 orders of magnitude.^114^^,^^118^^,^^119^^,^^120^^,^^121^^,^^122^

In conventional RS, molecular polarization (P_0_) primarily governs the Raman intensity. The magnitude of P_0_, corresponding to the Raman-shift in frequency (ω_R_), is predominantly influenced by the polarizability of molecular electrons (α_0_^R^) and the intensity of the incident electromagnetic radiation (E_0_) with a frequency of ω_0_.^123^ As depicted in Equation 1,

(Equation 1) $$P_{0}\left(\omega_{R}\right)={\alpha_{0}}^{R}\left(\omega_{0},\omega_{R}\right)E_{0}\left(\omega_{0}\right)$$

Under SERS conditions, the Raman dipole (P) is governed by the altered Raman polarizability (α^R^) and the amplified local electromagnetic field (E_loc_) generated near the surface of the metallic nanostructure. This enhancement arises from the synergistic contributions of electromagnetic and chemical enhancement mechanisms.^123^ As shown in Equation 2,

(Equation 2) $$P\left(\omega_{R}\right)=\alpha^{R}\left(\omega_{0},\omega_{R}\right)E_{\text{loc}}\left(\omega_{0}\right)$$

**Figure 4:**
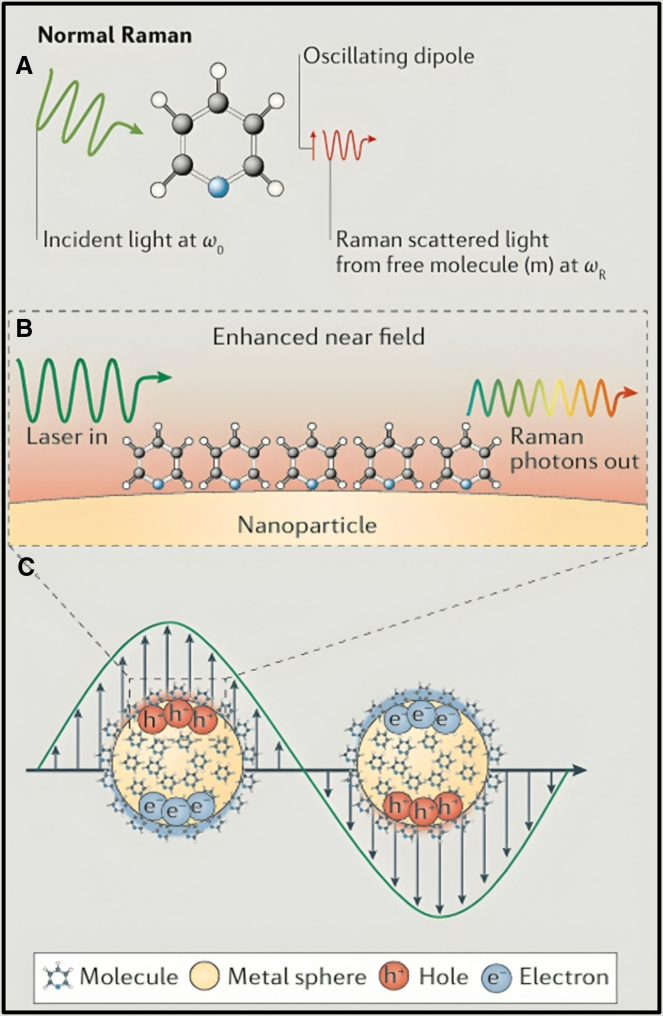
Principles of Raman and SERS Schematic representation illustrates the fundamental principles of (A) RS, (B) SERS, and (C) the underlying electromagnetic field enhancement. Reprinted with permission from Ding et al.^124^

The fundamental distinctions between RS and SERS are illustrated in Figures 4A–4C.

## Overview of the Raman and surface-enhanced raman spectroscopy techniques

### Raman spectroscopy for microplastics detection

RS is highly responsive to variations in the polarizability of chemical bonds, particularly in detecting functional groups such as aromatic bonds, C–H bonds, and C=C bonds. This sensitivity makes it a powerful tool for identifying and imaging MPs.^125^ Unlike infrared (IR) spectroscopy, RS is inherently insensitive to water, making it particularly advantageous for the analysis of MPs in aqueous and wet environments. Furthermore, RS has demonstrated effectiveness in determining the polymer types of MPs, including commonly encountered materials such as PE, PP, PET, and PS.^27^ In 2013, RS was employed to detect MPs in zooplankton samples^126^ published as the first report, establishing RS as a promising analytical tool for microplastic detection. Following this approach, in 2014, RS was utilized to study the uptake and retention of microplastics in shore crabs.^127^ Later, in 2015, several researchers adopted RS for the detection of microplastics in various environmental samples, highlighting its growing relevance in microplastic analysis.^26^^,^^128^^,^^129^^,^^130^ Further, in 2015, Collard et al. detected MPs using RS. The study showcased a process involving the digestion and isolation of MPs, followed by their detection using RS. From nine fish samples, they successfully isolated 11 MPs and 13 fibers composed primarily of cellulose and lignin, emphasizing the potential of RS as a powerful analytical tool for MP detection.^131^

**Figure 5:**
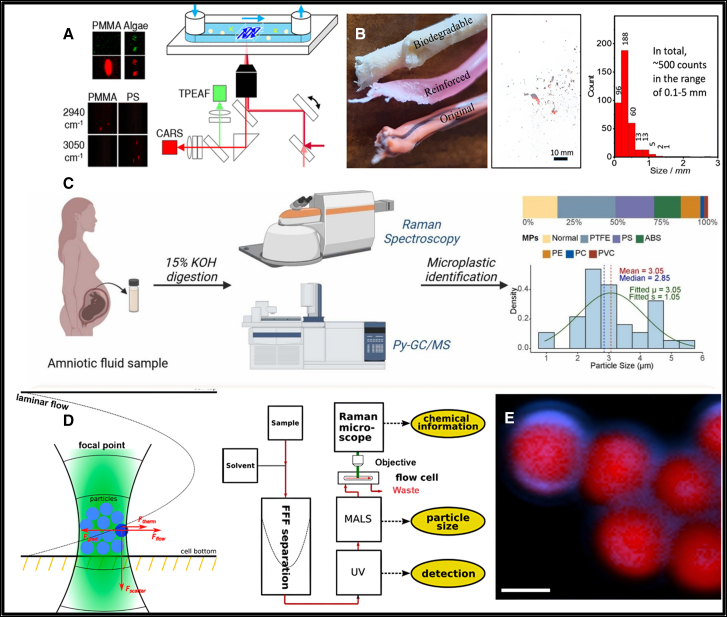
Raman techniques for microplastic detection (A) Schematic representation of the experimental setup for two-dimensional coherent anti-stokes Raman scattering (CARS) and two-photon excited autofluorescence (TPEAF) detection of MPs in a flow system^132^; (B) digital photographs of the “biodegradable,” “reinforced,” and “original” trimmer worn lines obtained from the trimmer factory, along with their corresponding size histograms^133^; (C) dual-method approach integrating RS and pyrolysis gas chromatography-mass spectrometry (Py-GC-MS) for identifying MPs in amniotic fluid^134^; (D) schematic of the optical trap within the downstream flow cell. The laser focal point at the bottom of the cell creates a two-dimensional trap that facilitates the accumulation of particles^46^; and (E) Raman image of microplastics (red) and surface biomolecules (blue) in freshwater. Scale bar: 2 μm.^135^ Reprinted with permission from Schwaferts et al.^46^; Takahashi et al.^132^; Luo et al.^133^; Tian et al.^134^; Ramsperger et al.^135^

In 2021, Takahashi et al. presented a novel approach for high spatial and temporal resolution two-dimensional identification and the classification of organic biotic particles and flowing MPs. This method relied on the simultaneous detection of CARS and two-photon excited autofluorescence (TPEAF) signals. Using this setup, they monitored algae and poly (methyl methacrylate) (PMMA) particles flowing at an average velocity of 1.17 mm/s using CARS scanning. The CARS signals at a vibrational frequency of 2940 cm^−1^ exhibited average intensities for both PMMA and algae particles that were distinctly higher than the background levels. However, TPEAF signals were exclusively emitted by algae, enabling the successful classification of PMMA and algae particles in flow.^132^ The experimental configuration for CARS and TPEAF detection is illustrated schematically in Figure 5A. Similarly, Levermore et al. have employed Raman spectral imaging combined with a chemometric analysis system to identify MPs with sizes ≥2 μm in ambient particulate matter. The study demonstrated the method’s applicability by successfully detecting airborne MPs.^29^ Later in 2022, Fang et al. employed Raman imaging to identify MPs and NPs released from trimmer lines during lawn mowing. Principal component analysis (PCA) and logic-based algorithms were utilized to achieve high-precision identification. Figure 5B presents the digital micrograph of MP generated from the trimmer, accompanied by the corresponding particle size distribution histogram.

Interestingly, RS offers the advantage of seamless integration with other analytical techniques, enhancing its versatility in complex studies. In this context, Tian et al. reported the first investigation utilizing a combination of RS and pyrolysis gas chromatography/mass spectrometry (GC/MS) to detect MPs in human amniotic fluid and assess their impact on pregnancy outcomes, as shown in Figure 5C. The study identified six distinct MPs types in samples from 39 subjects, with an average particle size of 3.05 ± 1.05 μm.^134^ A novel particle-capturing medium consisting of perfluoro hexane (PFH) in an L-shaped tube was developed by Cho et al. for the detection of PE MPs present in tap water, demonstrating a recovery rate of 95.9%.^136^ Furthermore, the hyphenation of a Raman spectrometer with a separation technique such as field-flow fractionation (FFF) was demonstrated by Ivleva et al. in 2021.^46^ To overcome the weak Raman scattering signal, a trapping strategy based on two-dimensional (2D) optical tweezers was employed. The model of the 2D optical trap and the hyphenation scheme are illustrated in Figure 5D. Wang et al. combined SERS with 3D imaging using SRS to investigate the composition of polymers in medical face masks. Their study successfully identified the release of inorganic additives, such as CaCO_3_, from the masks. The integration of these techniques revealed that microplastics released are complex mixtures of polymers and additives rather than pure polymers. This highlights the significant role of additives in accelerating photoaging and increasing microplastic emissions.^137^

Scientists have developed a wide-field hyperspectral Fourier transform Raman microscope for the rapid detection and identification of microplastics (MPs). This instrument uses a common-path birefringent interferometer, offering high spatial (∼1 μm) and spectral (∼23 cm^−1^) resolution while reducing sample fluorescence by adjusting the interferometer’s scan interval. After validation on commercially sourced MPs, the microscope demonstrated effective detection of MPs extracted from various matrices, including seawater and fish gastrointestinal tracts, making it suitable for large-scale environmental monitoring due to its speed (∼15 min for a 100 kpixel image), accuracy, and cost-effectiveness.^138^ Later, Prata and her coworkers demonstrated the effectiveness of pre-selecting suspected MPs using Nile Red staining in combination with a micro-Raman spectrometer. Using a 442 nm laser in micro-RS induced luminescence from Nile Red, enabling the targeted identification of suspected MPs particles when using an orange filter. This approach significantly reduced the number of particles requiring further analysis and enhanced sample throughput.^45^ Furthermore, the ingestion of microplastics by organisms due to environmental pollution was studied, revealing that biomolecules form an eco-corona on the surfaces of microplastic particles. Raman spectroscopic analysis of these surface coatings in freshwater was conducted.^135^ A false-color Raman image illustrating MP particles in red and surface-associated biomolecules (eco-corona) in blue, generated from spectral mapping data, is shown in Figure 5E.

Recent progress in computational methods has further enhanced the potential of RS. Innovations integrate deep learning techniques and sophisticated algorithms, facilitating automated and efficient spectroscopic data analysis. Such advancements have broadened their applications, improving data interpretation and accuracy.^139^ Contemporary advancements in RS have enhanced the detection and identification of MPs and NPs in diverse real-world samples. By comparing unknown particles against a comprehensive library of characteristic vibrational fingerprints, this technique enables the precise identification of MP compositions.

### Surface-enhanced raman spectroscopy for microplastics detection

SERS significantly enhances Raman signals by employing plasmonic metal nanoparticles.^140^ Due to its sensitivity, SERS is being widely employed for the detection of MPs, offering advantages such as simple sample handling, rapid analysis, and instrument portability.^14^^,^^141^^,^^142^^,^^143^ Furthermore, it facilitates non-invasive detection and supports single-molecule sensitivity with minimal sample preparation requirements.^144^

The limit of detection (LOD) for micro- and nanoplastics is influenced by various factors, including the choice of substrate template, instrument configuration, laser wavelength, nanoparticle properties (such as size, shape, and surface functionalization), sample concentration, matrix interferences, and pretreatment methods—all of which significantly affect the enhancement factor. Other contributing factors include the type and chemical composition of the plastic polymers, particle size and morphology, aggregation state of the particles, exposure time during measurement, environmental conditions (such as pH and ionic strength), and the presence of fluorescent or Raman-active background compounds that may interfere with signal detection. Microplastic detection via SERS leverages two key enhancement mechanisms: EM enhancement, arising from localized surface plasmon resonances (LSPR) in metallic nanostructures, and CE, which involves charge transfer between the plastic (or associated molecules/additives) and the substrate. For instance, Au@Ag nanostar arrays exploit the EM mechanism strongly due to their branched tip geometries and neighboring branch coupling, enabling the sensitive detection of small microplastics in water samples (e.g., Au@Ag NSs).^145^ In contrast, hybrid metal-semiconductor substrates such as Ag/ZnO@PDMS combine EM from Ag nanoparticles with CE contributions facilitated by ZnO, yielding both high sensitivity and enhanced reproducibility (RSD ∼5.9%) in multiple water matrices.^146^ Similarly, Co_3_O_4_/Co_3_S_4_ heterojunctions decorated with Ag NPs demonstrate that charge transfer from the semiconductor can boost detection performance, especially for smaller particles (∼100 nm), when paired with plasmonic metals.^147^

Several strategies have been employed to detect MPs using SERS. SERS substrate fabrication is an important step in efficiently detecting MPs. We discuss two primary approaches for fabricating SERS substrates: (i) colloidal and (ii) planar SERS substrates. SERS substrates have been designed to improve sensitivity by targeting key mechanisms, including (i) the effective trapping of MP particles, (ii) enhancing analyte concentration, and (iii) generating localized electromagnetic “hotspots”, each playing a vital role in enabling accurate and sensitive MP detection.

### Colloidal surface-enhanced raman spectroscopy substrates

**Figure 6:**
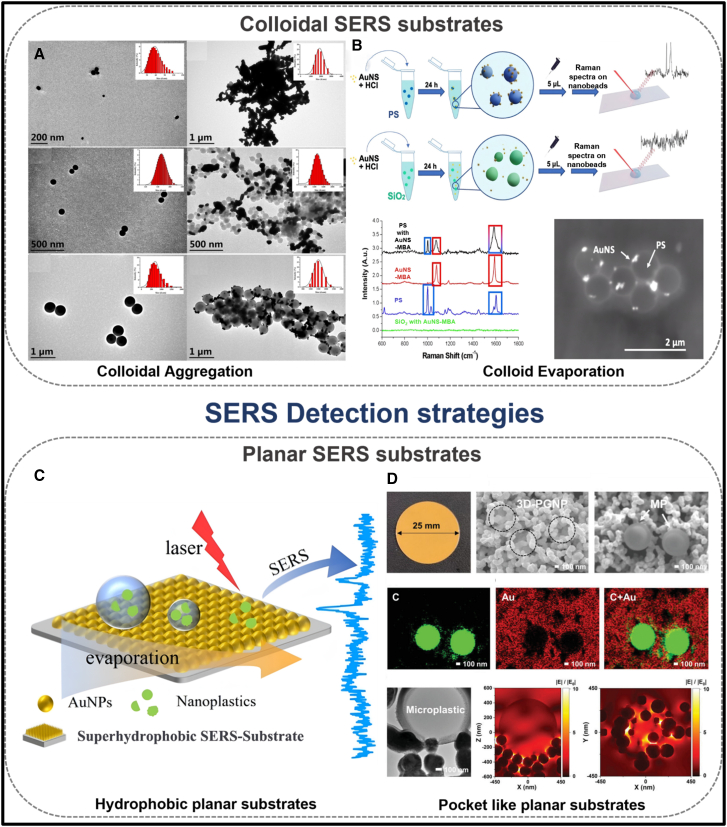
SERS substrates for micro/nanoplastics detection (A) TEM and DLS images depict the morphological and size characteristics of dispersed Ag nanoparticles, aggregated silver NPs, and dispersed PS spheres with diameters of 100 nm and 500 nm^148^; (B) Schematic of AuNS-MBA incubation with PS and SiO2 nanobeads, followed by μ-Raman analysis. Normalized Raman spectra of individual and incubated components were recorded on an Al foil to avoid glass interference. SEM backscattered electron image shows AuNS-MBA distribution on PS spheres^149^; (C) Schematic representation illustrates the detection of NPs using superhydrophobic substrates^150^; and (D) SEM image of 3D-PGNP and 3D-PGNP with a PS particle (1 μm), EDS images showing the carbon map, Au map, and the merged carbon-Au map of the PS particle, TEM image of 3D-PGNP with PS particle inclusion, and FDTD simulation of 3D-PGNP with PS particle inclusion.^151^ Reprinted with permission from Lv et al.^148^; Mercedi et al.^149^; Xing et al.^150^; Kim et al.^151^

Colloidal substrates, constituting nanoparticles dispersed in liquid analytes, provide an extensive surface area that facilitates efficient molecule-substrate interactions, thereby enhancing SERS signals with high reproducibility. In 2020, Lulu and colleagues developed an aggregation strategy using NaCl to enhance the density of “hot spots” for detecting PS, PE, and PP in pure and seawater samples. They optimized the NaCl concentration to 0.25 mol/L. It was noted that with increasing salt concentration, aggregation became more pronounced, preventing silver nanoparticles from binding to the surface of 100 nm PS spheres. This approach demonstrated a significant enhancement factor of up to 4 × 10^4^, indicating high sensitivity for detection. The TEM and dynamic light scattering (DLS) images of dispersed and aggregated Ag nanoparticles are presented in Figure 6A.^148^ These images show that higher NaCl concentrations lead to aggregation of PS spheres (100 and 500 nm), which become decorated with silver nanoparticles after mixing. The resulting dense clusters on their surfaces generate abundant SERS hot spots, thereby enhancing the Raman signals.^148^

Additionally, the detection of NPs was accomplished through an evaporation-based strategy, wherein a suspension containing plasmonic nanoparticles and NPs was deposited onto a silica wafer.^28^ This process resulted in a concentrated composite of NPs and nanoparticles.

The prepared substrates were analyzed via Raman mapping, demonstrating the efficacy of this approach.^28^ Later, in 2024, the detection of MPs using SERS-tagged AuNSs was demonstrated by Litti and his research group.^149^ The approach utilized the strong intrinsic signals from the SERS tags as spectral fingerprints to identify both MPs and NPs. The method’s applicability was validated through the successful detection of MPs in offshore marine samples.^149^ A schematic representation of the incubation strategy employing PS beads, along with detection using 4-MBA as a Raman reporter and corresponding SEM images, is presented in Figure 6B.

### Planar SERS substrates

Planar substrates, comprising solid surfaces, serve as platforms for fabricating nanostructures through nanoparticle deposition or self-assembly processes, effectively generating localized hotspots for analyte adsorption.^152^ Their scalability is enhanced by large-scale production techniques, including thin-film deposition,^153^ lithography,^154^ and nanoimprinting,^155^ making them viable for commercial applications.^156^ In 2020, Xu et al. utilized the commercially available Klarite substrate, characterized by a dense array of gold-coated, inverted pyramidal cavities. This substrate exhibited notable potential for the detection and identification of MP particles as small up to 360 nm. Their investigation reported enhancement factors reaching up to two orders of magnitude for PS analytes.^2^ The application of the Klarite substrate for MPs detection. In 2025, Fan and his research group demonstrated the detection of NPs using superhydrophobic substrates fabricated via a liquid-liquid self-assembly method. In their study, PS nanoparticles ranging from 30 to 1000 nm were detected in water samples, while PET was identified in bottled water.^150^ A schematic illustration of the superhydrophobic substrate is presented in Figure 6C. Similarly, the fabrication of substrates resembling capturing pockets integrated with plasmonic nanoparticles has exhibited notable signal enhancement and impressive sensitivity. In this regard, Kim et al. developed a 3D-plasmonic gold nanopocket nanostructure (3D-PGNP) on filter paper substrates for the detection of PS MPs.^151^

Furthermore, they utilized machine learning (ML) techniques to quantitatively analyze the MPs in the samples, providing a robust approach for accurate detection and quantification.^151^ Figure 6D presents the TEM, SEM, and EDS mapping analyses, alongside Finite-Difference Time-Domain (FDTD) simulations, for characterizing the 3D nanopockets. Table 2 summarizes the detection of micro- and nanoparticles using SERS and RS, detailing the methods employed, concentration ranges, instrumentation used, real sample analyses, enhancement factors, advantages, and measured plastic sample sizes.

**Table 2:** Summary of SERS and RS studies for the detection of micro- and NPs, highlighting particle size and real sample analysis

Raman/SERS	Platform/Template	Methodology	Instrument	Nature of the plastics	Size of the plastics	Concentration	Real sample analysis	Naturally available/spiking	LOD	Enhancement factor	Advantages	Limitation
Raman^157^	Filter membrane	Raman tweezers	Raman microscope (532 nm)	Not mentioned	1 - 5000 μm	Qualitative	Crab and fish	Naturally available	–	–	Detecting plastics from the real sample matrices	**Detection size of MP is broader**
Raman^158^	Aluminum oxide filters	Filtration	Micro Raman spectrometer (442 & 633 nm)	PE	122 to 26 μm	2,563–5857 particles. L^−1^	White wine	Naturally available	–	–	Detecting plastics from the real sample matrices	**Quantification was done using staining and needs microscopic evaluation**
SERS^148^	Cuvette	Ag colloid with NaCl aggregates	Portable Raman spectrometer (785 nm)	PS, PE, PP	100 nm, 500 nm, and 10 μm	40 μg/mL	Pure water, seawater	Spiking	100 nm plastics up to 40 μg/mL	4 × 10^4^	Different sizes of MP are detected up to nm	**Plastic particles are synthesized using a chemical method**
SERS^2^	Klarite	Deposition	Confocal Raman spectrometer (785 nm)	PS, PMMA	450 nm	2.625 × 10^−5^ g/cm^−3^	Air sample	Naturally available	Single micro-/nanoplastic particles down to 360 nm	≈10^2^	Capturing of air samples	**Matrix interference**
SERS^159^	Glass microscopy slides	Spherical Au nanoparticle – 14 and 46 nm	Confocal Raman spectrometer (785 nm)	PS, PET	616, 33, and 62 nm	10 μg/mL	–	–	10 g/mL	Analytical enhancement factors of up to 446	Low LOD	**No real sample analysis**
SERS^28^	Silicon wafer	Ag colloid, PS, and MgSO_4_ mixture	Raman microscope (532 nm)	PS	1 μM and 50 nm	10 μM	River water	Spiking	5 mg/L	–	NPs detection	**Uneven distribution of NPs in Si wafer**
SERS^142^	AAO template	Anisotropic nanostar dimer-embedded nanopore	Confocal laser micro-RS	PS	0.8 μm, 2.3 μm, and 4.8 μm	∼0.05 mg/g 50 ppm (0.4 μm)	DI water, sea, tap, and river water	Spiking	0.005% by weight (≈0.05 mg plastic per g substrate/sample) for the 0.4 μm PS microplastic spheres	–	Low LOD	**Complicated fabrication procedure**
Sers^151^	23days-PGNP nanoarchitecture on Ca filter paper	Direct gold nucleation	Portable Raman spectrometer (785 nm)	PS, and PE	PE: 1–4 μm	5.3 μgml^−1^	DI, tap, river, and seawater	Spiking	–	–	Detected a mixture of plastics and low-level detection	**No real sample analysis**
SERS^160^	Tin foil	Simple citrate chemical reduction	Confocal Raman spectrometer (785 nm)	PS	20 nm	0.0005% of 360 nm PS	Food packaging boxes	Naturally available	0.0005% by weight (≈5 parts per million by weight) in water	–	Easy sample preparation and real sample analysis	**No real sample analysis**
SERS^161^	Si wafer	Microwave synthesizing and chemical reduction	Portable Raman spectrometer (785 nm)	PVC, PS, and PVA	PVC - 0.624 g/mL to 0.624 ng/mL, PVA - 0.4 g/mL to 1.1 mg/mL, and PS - 26 mg/mL to 26 μg/mL	PVC - 0.1 nM and 1 nM, PVA - 10 mM, and PS - 10^−6^ M	Tap water and river water	Spiking	–	–	Ultralow detection of MPs in water	**No real sample analysis**
SERS^162^	Filter paper	Simple syringe filtration method	Raman microscope (532 nm)	PSNSs	100, 300, 460, 600, and 800 nm	0.31 μgmL^−1^	Bottled drinking water, tap water, and river water	Spiking	0.31 μg mL^−1^ for PSNSs in water	–	Good selectivity	**No real sample analysis**
SERS^163^	Silicon or glass wafer coated with gold	Drop-casting on a silicon or glass wafer coated with gold	Micro-Raman spectrometer connected to a Leica microscope (785 nm)	PS	350 nm	6.25 μgmL^−1^	–	–	6.5 μg/mL for the 350 nm PS spheres in pure water	–	Low-level detection	**No real sampleanalysis**
SERS^63^	Macro-mesoporous Ag foams coated with a hydrophobic layer	Deposition	JASCO NRS3100 spectrometer (532 nm)	PS	0.36 to 5 μm	0.0015 mgL^−1^	Environmental samples, wastewater, seawater, and samples containing humic acid	–	∼10 MP L^−1^	2.5×10^5^	Stable substrate and multiple detection	**No real sample analysis and complex sample preparation**
SERS^143^	Regenerated Cellulose with Ag nanowires	Simple vacuum-assisted filtration method using a silicon mask	Not mentioned (785 nm)	PS	84, 444, and 630 nm	1 mg/mL	No	No	0.1 mg/mL	1.8 × 10^7^	Flexibility, and NPs detection	**No real sample analysis**

## SERS enhancement mechanisms and substrate design optimization

SERS signal amplification primarily arises from EM and CE, with nanoparticle morphology—such as shape, size, and interparticle spacing—playing a critical role. Sharp-tipped structures such as gold nanostars or silver nanowires generate intense hot spots; optimal particle sizes (∼50–100 nm) balance plasmon resonance and field localization, while interparticle gaps of 2–5 nm maximize EM coupling without causing quenching. Surface functionalization (e.g., thiol or hydrophobic modifications) further improves analyte adsorption, stability, and selectivity toward hydrophobic micro/nanoplastics. Both experimental studies and theoretical simulations confirm that these design parameters directly impact SERS enhancement factors (up to 10^6^), reproducibility, and sensitivity, enabling the detection of microplastics and nanoplastics at concentrations down to 0.1 μg/mL.^164^^,^^165^ A study advances the quantitative detection of trace nanoplastics (<100 nm) using a hydrophobic CuO@Ag nanowire substrate combined with a multiplex-feature analysis based on the coffee ring effect. The optimized substrate enhances Raman signals via abundant plasmonic hot spots and analyte enrichment. Integrating Raman intensity, coffee ring size, and detection probability with ML enables ultralow LODs (10^−10^wt %) and 19-fold improved accuracy over traditional methods. This approach offers sensitive, rapid, and precise nanoplastics detection for environmental and biological applications.^166^ Optimized substrates with controlled morphology and surface chemistry, therefore, provide a robust platform for sensitive, reproducible detection of diverse micro- and nanoplastic particles, highlighting the critical role of rational substrate design in SERS-based environmental monitoring.

## Size-dependent detection of micro- and nanoplastics using SERS

Microplastics (MPs) and nanoplastics (NPs) exhibit a broad size distribution, which significantly influences their detection sensitivity and reliability. SERS has shown great promise for the rapid and trace-level identification of these particles, with substrate characteristics—such as nanoparticle shape, size, spacing, and surface modification—playing a critical role in signal enhancement and reproducibility. Table 3 classifies microplastics by particle size and lists the corresponding SERS substrates and enhancement mechanisms used for their detection.^167^ Several studies have demonstrated the size-dependent detection of microplastics (MPs) and nanoplastics (NPs) using SERS substrates. Shorny et al. imaged and identified single nanoplastic particles down to 100 nm using SERS combined with confocal microscopy, demonstrating the value of spatially resolved SERS for small particles.^168^ Patterned gold films with high-density nanogaps have been used to detect 50 nm polystyrene nanoparticles (PS) at concentrations down to ∼1 μg mL^−1^ and to identify ∼100 nm PP/PET particles in filtered drinking/wash water.^24^ Gold-nanostar SERS substrates have been applied across multiple polymer types and sizes, enabling the detection of sub-100 nm PS (e.g., 33 nm) and small PET particles at μg·mL^−1^ levels, which highlights polymer-dependent differences in analytic sensitivity.^165^ Substrate engineering that concentrates analyte (for example, superhydrophobic CuO@Ag or Ag-decorated CuO nanowire arrays) markedly improves enrichment and hot-spot density and thus enhances detection for smaller plastics.^169^ These findings collectively demonstrate that both SERS substrate design and particle size play pivotal roles in determining detection sensitivity, recovery efficiency, and the practical applicability of the technique in complex environmental matrices.

**Table 3:** The table presents a classification of microplastics based on particle size, alongside the corresponding SERS substrate types and their associated enhancement mechanisms used for detection

**Target Microplastic Size**	Substrate Type	Materials	Mechanism
Large Microplastics (1 mm to <5 mm)^77^	Large Microplastics (1 mm to <5 mm)	Conventional RS (without SERS)	These particles are large enough to generate a strong Raman signal on their own, making SERS enhancement unnecessary.
All sizes, but particularly effective for small microplastics and nanoplastics (down to 33 nm)^148^^,^^164^^,^^165^	Colloidal Nanoparticles	Gold (Au) or Silver (Ag) nanoparticles in a solution	The nanoparticles are mixed with the sample and aggregate around the plastic particles, creating plasmonic “hot spots” that amplify the Raman signal.
Small microplastics and nanoplastics (down to 50 nm)^142^^,^^167^	Nanostructured Thin Films	Gold or Silver films on a solid support (e.g., silicon)	The film is patterned with nanostructures such as pyramids, rings, or pores, which serve as pre-defined hot spots. The plastic particles are drop-cast onto the substrate.
Submicron- and nanoplastics (down to 33 nm). Particularly useful for low concentrations.^164^^,^^165^	Gold Nanostars	Star-shaped gold nanoparticles with multiple spikes	The sharp points of the nanostars act as highly concentrated hot spots, providing a strong SERS signal.
Nanoplastics (down to 1 nm in some studies)^155^	Composite Substrates	Combinations of materials, such as Au-Ag composites or metal-semiconductor arrays	These substrates combine the plasmonic properties of metals with other materials to improve sensitivity and signal uniformity.

## Emerging detection strategies and benchmark metrics for micro- and nanoplastics

MPs and NPs have emerged as growing pollutants due to their non-biodegradability and small size, posing significant health risks and ecological disruption. SERS has gained attention as a potential analytical tool for its detection, offering high sensitivity and molecular specificity. Recent case studies, coupled with ML-assisted spectral analysis and benchmark datasets, are accelerating the translation of SERS toward regulatory validation and large-scale field monitoring. In 2025, Caldwell et al. developed a gold nanostar-based SERS substrate that enabled sensitive detection of plastics below conventional resolution limits, including 33 nm polystyrene (1.25 μg mL^−1^) and 36 nm PET (5 μg mL^−1^), along with polypropylene and polyethylene NPs. This demonstrated the feasibility of detecting submicron (1 μm-100 nm) and NPs (<100 nm), which remain challenging due to their small size and low environmental concentrations.^165^ In another study, researchers used SERS imaging to identify single NPs as small as 100 nm and to distinguish them from agglomerates, achieving a 10^7^-fold improvement in detection speed compared to conventional SERS imaging. Resolving single particles allowed precise measurement of enhancement factors, advancing nanoscale plastic sensor development. This bridges the gap between bulk NP analysis and deployable SERS sensors capable of quantifying NPs presence in real environments.^168^ Ahn and co-workers further implemented a SERS-based method using surface acoustic waves (SAWs) to generate gold nanogaps on PS MPs for detecting adsorbed toxic substances. Polycyclic aromatic hydrocarbons (PAHs), including pyrene, anthracene, and fluorene, were detected with limits of detection of 95, 168, and 195 nM, respectively. A strong linear correlation (R^2^ = 0.98) was observed between SERS intensity and PAH concentration. This method enabled the multiplex detection of PAHs on microplastic surfaces, offering a tool for monitoring pollutant-microplastic interactions in aquatic systems.^170^ Kumar et al. (2023) used AgNP-decorated substrates fabricated via a toluene dispersion and evaporation-induced self-assembly (EISA) strategy for PS, PP, and polyvinyl chloride (PVC) detection, achieving LODs down to 0.001 mg/mL and demonstrating strong linearity (R^2^ = 0.986–0.995) across aqueous and real-world samples.^171^

## Separation and enrichment strategies for reliable SERS-based detection of microplastics

Effective separation and enrichment are essential prerequisites for reliable SERS detection of micro- and nanoplastics, as direct analysis of environmental matrices is often hindered by low particle concentrations, matrix interferences, and heterogeneous polymer compositions. High-gradient magnetic separation (HGMS) has been reported to achieve recovery rates above 90% for diverse polymer types in soil matrices, though performance declines for particles smaller than ∼60 μm.^172^ Similarly, affordable density separation devices (DSDs) employing sodium polytungstate as a flotation medium demonstrated >78% recovery for fragments >100 μm in deep-sea sediments, with near-complete recovery for PVC and high-density polyethylene.^173^ Additionally, surfactant-assisted density separation using non-ionic agents such as Tween 20 has been shown to improve separation purity in mixed polymer systems, achieving up to 97 ± 2% purity for PET.^174^ Collectively, these strategies demonstrate significant progress in achieving high recovery and reproducibility; however, the enrichment of particles below 100 nm and in complex, heterogeneous samples remains a critical challenge.

## Detection of microplastics in real-world samples

Micro- and NPs are commonly found in diverse environmental samples, including freshwater, seawater, sediments, food items, and biological tissues.^175^^,^^176^^,^^177^ These samples often contain mixed matrices of organic and inorganic contaminants. MPs exhibit significant heterogeneity in terms of shape, size, and composition. Therefore, before their identification and quantification, pretreatment methods are crucial to eliminate matrix interferences in real-world samples. A few key pre-treatment strategies include: (i) preconcentration - enhancing the detectability of MPs by increasing their relative concentration, (ii) separation - isolating MPs from other particulates, and (iii) matrix digestion - removing organic or inorganic materials that obscure detection. Such preparatory steps are vital for ensuring accurate and reliable analysis of MPs in complex matrices.

Preconcentration techniques, including cloud point extraction,^178^ continuous flow centrifugation,^179^ ultrafiltration,^180^ and ultracentrifugation,^181^ serve as efficient approaches to enhance the LOD and limit of quantification (LOQ) in analytical methodologies.^182^ For example, ultrafiltration utilizing a polyether sulfone (PES) - based membrane system has been applied to concentrate and separate NPs from samples with relatively minimal impurities, such as seawater and drinking water.^182^ The retained fraction obtained post-ultrafiltration, exhibiting an enrichment factor of up to 500, was subsequently collected for detailed characterization.^183^ Similarly, size-based separation is a widely adopted method for isolating particles in various samples, employing techniques such as FFF, SEC, and filtration methods. Ter Halle et al. detected NPs using a 1.2 μm pore-sized PES membrane in the North Atlantic Subtropical Gyre to filter out larger particles before the pre-concentration step.^182^ A comparable pre-treatment approach was reported in a study on NPs in wastewater, which utilized a 1 μm pore-sized glass fiber membrane.^183^

For accurate analysis, it is crucial to isolate MPs from environmental matrices. Numerous digestion techniques have been developed for this purpose in MPs studies.^184^ Common approaches include acidic treatments, such as 65% nitric acid^185^ and 30% hydrogen peroxide, as well as alkaline treatments with sodium hydroxide or potassium hydroxide,^186^ which effectively degrade organic matter and facilitate particle separation. Moreover, enzymatic digestion protocols, known for their gentle and non-destructive nature, have been tailored for tissue digestion and are increasingly applied in micro/NPs research.^187^

**Figure 7:**
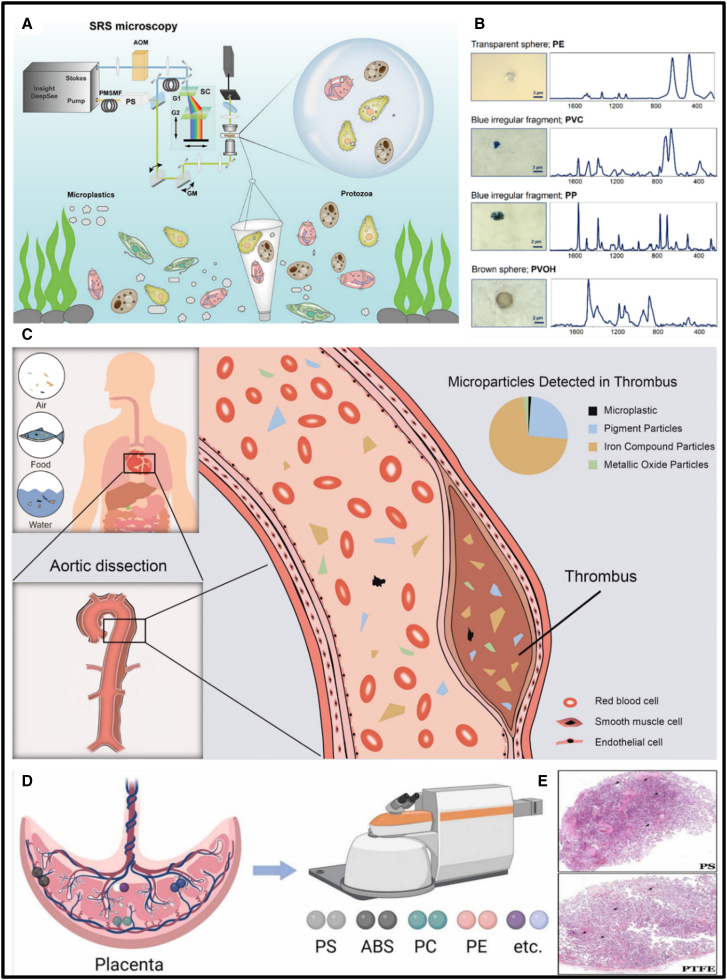
Raman and SERS detection in real samples (A) Schematic representation of stimulated Raman scattering (SRS) microscopy for quantifying the bioaccumulation of small MPs^188^; (B) Microphotographs and corresponding Raman spectra of selected MPs, including PE, PVC, PP, and PVA, identified in analyzed breast milk samples^16^; (C) Schematic representation of the detection of the microplastics and the pigment microplastics in the human thrombi using RS^189^; and (D) Application of confocal RS for the detection of MPs such as PTFE, PS, and ABS in human placenta samples^190^ and (E) Placental pathological changes associated with PTFE and PS exposure.^190^ Reprinted with permission from Ragusa et al.^16^; Wang et al.^188^; Wu et al.^189^; Yun et al.^190^

Several investigative studies have reported the detection of MPs and NPs in real-world samples, providing critical insights into their environmental and biological impacts. Wang et al. recently applied SRS microscopy to detect and measure the accumulation of MPs smaller than 10 μm within protozoa. This study, illustrated in Figure 7A, demonstrated the detection of PE, PP, PVC, PET, PS, and PMMA. Protozoa in water samples collected from the Yangtze River, Xianlin Wastewater Treatment Plant, Lake Taihu, and the Pearl River Estuary accumulated MPs smaller than 10 μm. However, the proportion of cells containing MP was relatively low, ranging from approximately 2%–5%.^188^ Similarly, the accumulation of MPs in human breast milk was investigated by Ragusa and co-workers in 2022 using Raman micro-spectroscopy. Breast milk samples from 34 women were analyzed, revealing MP contamination in 26 of 34 samples. This study is the first to demonstrate the microphotographs and Raman spectra of MPs detected in human breast milk, including PE, PVC, PP, and polyvinyl alcohol (PVOH), as shown in Figure 7B. The characterized MPs were systematically grouped by morphology, color, size, and molecular structure.^16^ In 2022, Zhang and coworkers developed NIR SERS-labeled nanoparticles for the ultrasensitive detection of NPs at the single-particle level in zebrafish embryos. The study involved exposing zebrafish embryos and larvae to nanoparticles, followed by RS analysis. Raman intensity mapping and images demonstrated the targeted localization of nanoparticles in embryos at 36 h postfertilization (hpf) and in larvae at 48–120 h postfertilization (hpf), with exposure durations ranging from 0 to 48 h.^191^

In 2023, Luo, Xia, and co-workers investigated the presence of pigmented microparticles and microplastics in human thrombi using RS. The detected particles exhibited block-like morphologies and varied in size from 2.1 to 26 μm, with approximately 69% measuring below 26 μm.^189^ The authors highlighted that the complex biological matrix of thrombi—rich in proteins, lipids, and other organic constituents—can lead to significant spectral overlap and fluorescence background, thereby complicating the accurate identification of microplastics and pigments. To address these challenges, a comprehensive pretreatment strategy was employed, involving enzymatic digestion and chemical treatments to effectively remove organic matter. This approach significantly enhanced the specificity and sensitivity of Raman spectral analysis. A schematic illustration of the detection workflow and the composition of the identified particles is provided in Figure 7C.^189^ Similarly, Yun et al. (2024) employed confocal RS with a detection threshold of ∼0.25 μm to identify microplastics (MPs) such as PTFE, PS, and ABS in human placental tissue. Despite the biological matrix being rich in proteins, lipids, and other biomolecules that can cause background fluorescence and spectral overlap, the authors applied data-processing techniques, including principal component analysis (PCA), to minimize noise and enhance detection accuracy. Among the 50 placenta samples analyzed, 31 were found to contain a total of 40 MPs, with particle sizes ranging from 1.03 to 6.84 μm. The identified polymers were predominantly PTFE, PS, and ABS, as illustrated in Figure 7D. Although MPs were present, histological examination using hematoxylin and eosin (H&E) staining revealed no significant pathological alterations in placental tissues, even in samples exposed to PTFE and PS, as shown in Figure 7E.^190^

## Matrix interference and standardized protocols in real-world SERS analysis of microplastics

The detection of microplastics (MPs) in real-world samples is complicated by matrix interferences from salts, humic substances, proteins, and biofilms, which can overlap or suppress Raman signals, reducing sensitivity. Natural organic matter, dyes, and biofilms often cause strong fluorescence that obscures characteristic polymer peaks, especially in colored or polymer-rich samples. Pretreatment methods such as filtration, density separation, solvent rinses, sieving, size fractionation, drying, concentration, and chemical or enzymatic digestion can mitigate these effects; however, they may also cause polymer loss, size bias, or surface alteration, compromising reproducibility. A major challenge arises from fluorescent additives such as pigments and dyes that mask polymer signals. Liu et al. (2023) addressed this by applying a “sunlight-Fenton” treatment (Fe^2+^ at 1 × 10^−6^ M and H_2_O_2_ at 4 M for 14 h), which effectively degraded fluorescent components without requiring spectral post-processing. Among four catalysts tested, Fe^2+^ performed best, enhancing Raman spectral clarity of MPs from mangrove samples, with matching degrees exceeding 70% across various colors and shapes, demonstrating robustness in real-world MP analysis.^192^

**Figure 8:**
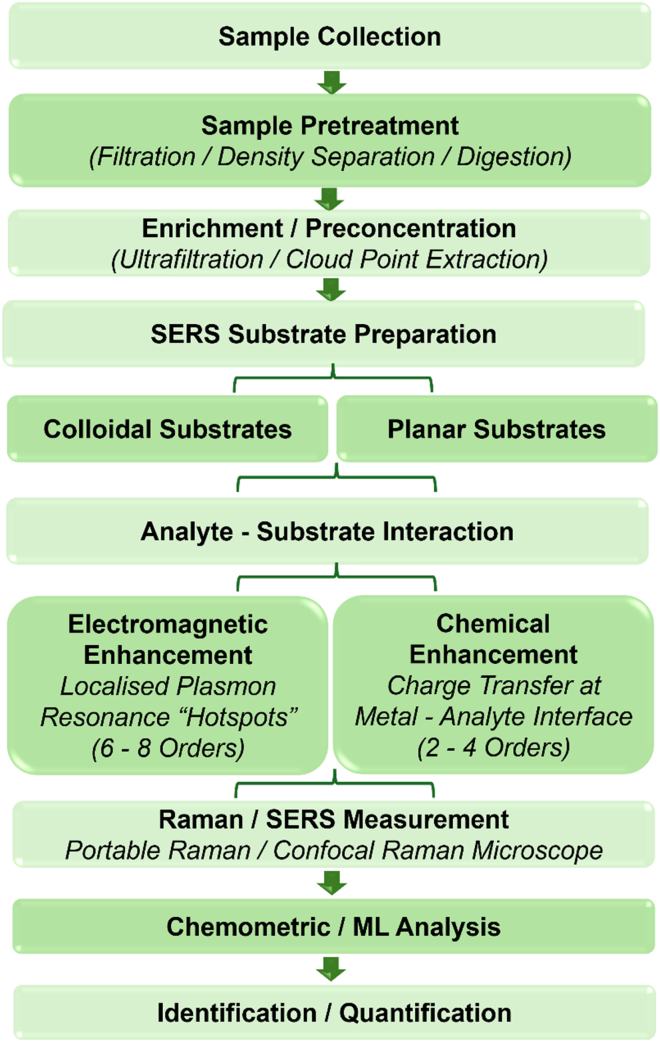
Workflow illustrating the key steps in SERS-based micro- and nanoplastics detection, from sample collection and substrate preparation to Raman measurement and data analysis

Additionally, RS often struggles to distinguish MPs from micro-additive particles due to their highly similar spectral signatures. Li et al. (2023) showed that slip additives commonly found in plastics have hit quality index values between 0.93 and 0.96—well above the 0.70 threshold typically used to identify MPs—leading to potential misclassification. They introduced an alcohol pretreatment protocol that effectively removes additive particles, enabling accurate MP identification in polyethylene pellets, bottle caps, and polypropylene containers. They also noted that additive particles can adsorb onto MPs, potentially increasing health risks. This cost-effective pretreatment offers a practical way to reduce additive interference and improve MP detection reliability.^193^ The overall workflow for SERS-based micro- and nanoplastics detection, including sample collection, substrate preparation, Raman measurement, and data analysis, is illustrated in Figure 8.

## Integration of Raman spectroscopy and machine learning for real-world microplastic detection

Microplastics often exhibit complex molecular structures and chemical compositions, which can make it challenging for researchers to distinguish them based solely on spectral features. To address these difficulties, ML has become an important tool for MP and NP detection. ML models can be trained to overcome fluorescence background, reduce spectral noise, and enhance the reliability of analysis, particularly in real environmental samples. Consequently, many researchers have increasingly adopted chemometric approaches such as principal component analysis (PCA), partial least squares discriminant analysis (PLS-DA), k-nearest neighbors (KNN), and support vector machines (SVMs) to improve the classification and identification of MPs and NPs. Recently, Li et al. integrated RS and Attenuated Total Reflection Fourier Transform Infrared Spectroscopy (ATR-FTIR) to classify eight types of microplastics using a 1D-convolutional neural network (CNN) with a multi-head attention mechanism, achieving 73% and 75% accuracy for ATR-FTIR and Raman, respectively. A three-level data fusion strategy improved classification to 88% (low-level), 97% (mid-level), and 99% (high-level). Validation in spiked samples (milk, Coke, tap water) showed the high-level fusion model maintained over 98% accuracy, demonstrating strong robustness and generalization.^194^ Researchers applied RS coupled RS with ML analysis to introduce a sensitive detection and classification of seven different types of MPs and analysis of real environmental samples. PCA and LDA enabled the clear separation of spectral signatures, while SVM classification achieved over 98% accuracy for most polymers and real-world samples, such as snack containers, bottled water, juice bottles, and medicine vials, which were accurately identified, with overall sensitivity, specificity, and accuracy of 98.1%, 99.4%, and 99.1%, respectively. Even after exposure to environmental stressors, the method remained effective, with SVM maintaining 96.75% accuracy, highlighting its reliability for automated microplastics detection.^195^

## Challenges and future perspectives

RS, as a robust analytical technique, has garnered significant attention for its ability to detect MPs both qualitatively and quantitatively. Along this direction, SERS further enhances the detection sensitivity of the Raman signals by leveraging plasmonic nanoparticles to amplify the signal intensity. Although the technique offers numerous advantages, it does have certain limitations, including challenges in selectivity, the requirement for extensive sample pretreatment, and other associated drawbacks. Before delving into the future directions, we enumerate the key challenges here.

1. **Signal Variability Due to MPs Characteristics**: In microplastic analysis, one of the notable shortcomings of SERS is signal variability caused by differences in the shape, size, and composition of MPs. These variations affect Raman enhancement, resulting in inconsistent signals, even at similar concentrations. Additionally, most studies have focused on qualitative detection, with limited efforts toward recovery studies or quantitative analysis. Most of the reported work focused on the commercially available microplastics in uniform size and shape.
2. **Detection of Colored MP Samples:** Environmental samples often include MPs of diverse colors and optical properties, which pose a challenge for SERS analysis. The choice of laser wavelength becomes critical to mitigate issues such as fluorescence noise and quenching effects. Optimizing the laser wavelength for such colored samples is essential to improve the accuracy and reliability of SERS detection. Another significant challenge is that SERS cannot detect all types of plastic materials. Certain plastics, such as fluoropolymers such as polytetrafluoroethylene (PTFE; Teflon) and polyvinylidene fluoride (PVDF), exhibit inherently weak Raman signals due to the low polarizability of their strong C-F bonds. Additionally, polyolefins such as PE and PP often exhibit weak and broad Raman peaks compared to aromatic polymers. Therefore, there is a need to develop SERS-based analytical methods capable of detecting a wide range of plastics, including those with weak Raman activity, in various shapes and sizes.
3. **Lack of Standardized Protocols and Spectral Libraries:** At this stage, SERS lacks standardized protocols for sample preparation, measurement, and data analysis for MPs analysis. The absence of a comprehensive spectral library for real-world MP samples further limits its widespread application and reproducibility in environmental studies.
4. **Tunable Substrate Architectures:** Hotspot engineering is crucial for advancing SERS substrates capable of detecting micro- and NPs. For effective quantitative detection, plastic particles must be positioned near accessible plasmonic hotspots. However, buried hotspots, where the hotspot volume is smaller than the NPs, remain inaccessible, leading to signal loss. This challenge is further exacerbated in the case of NPs, which often exhibit weak Raman signals and limited interaction with the substrate due to their extremely small size and low surface affinity. This issue could be addressed by fabricating substrates with exposed hotspot arrays of plasmonic nanoparticles, ensuring consistent interaction with the analyte.
5. **Challenges and Opportunities in Nanoplastics (<1 μm) Detection Using SERS:**
  a. Detecting nanoplastics—particles smaller than 1 μm—presents unique challenges due to weak Raman scattering signals, pronounced aggregation behavior, and complex interactions with SERS substrates. These factors can lead to weak and inconsistent signals, complicating reliable identification and quantification. Recent advancements have focused on developing substrates that enhance the SERS signal for nanoplastics. For instance, gold nanostar-based substrates have been utilized to detect various plastic types in the submicron and nanometer ranges, achieving detection limits as low as 625 ng/mL.^165^ Additionally, silver nanowire meshes have demonstrated the capability to detect 50-nm polystyrene nanoparticles at concentrations as low as 0.1 μg/mL.^24^
  b. Nanoplastics often exhibit aggregation behavior, which can lead to heterogeneous “hot spots” on SERS substrates, resulting in variable signal intensities. Moreover, interactions between nanoplastics and substrates can affect the reproducibility and uniformity of SERS signals. Addressing these issues requires the development of substrates that can effectively capture and stabilize nanoplastics, ensuring consistent signal enhancement.
  c. Studies have reported detection limits for nanoplastics in the range of 0.1 μg/mL to 625 ng/mL, depending on the substrate and particle size.^24^ Recovery rates for nanoplastics can vary, with some methods achieving rates as high as 94.1% in complex matrices such as drinking water. However, these rates can be influenced by factors such as particle size, aggregation state, and matrix composition.
  d. SERS offers several advantages over traditional techniques such as FTIR and pyrolysis-GC-MS for nanoplastic detection. It provides high sensitivity, minimal interference from water, and the potential for single-particle detection. Additionally, SERS can be coupled with advanced imaging techniques to visualize and identify individual nanoplastic particles, facilitating detailed analysis of their distribution and behavior.^196^
  e. Despite its advantages, SERS faces limitations in nanoplastic detection, particularly for particles in the 20–100 nm range. Challenges include substrate reproducibility, signal variability due to particle heterogeneity, and matrix effects. Future research should focus on optimizing SERS substrates to enhance signal uniformity, developing standardized protocols for sample preparation and analysis, and integrating SERS with other analytical techniques to improve detection capabilities.
6. **Challenges in Real Sample Analysis**: Real-world sample analysis introduces additional complexities, such as matrix interferences and the tedious nature of pre-treatment steps. Processes such as filtration, concentration, and particle selection are time-consuming and prone to errors, as small, transparent, or translucent particles might be overlooked. Simplifying and automating these steps is essential to make SERS a more practical tool for environmental analysis.

**Figure 9:**
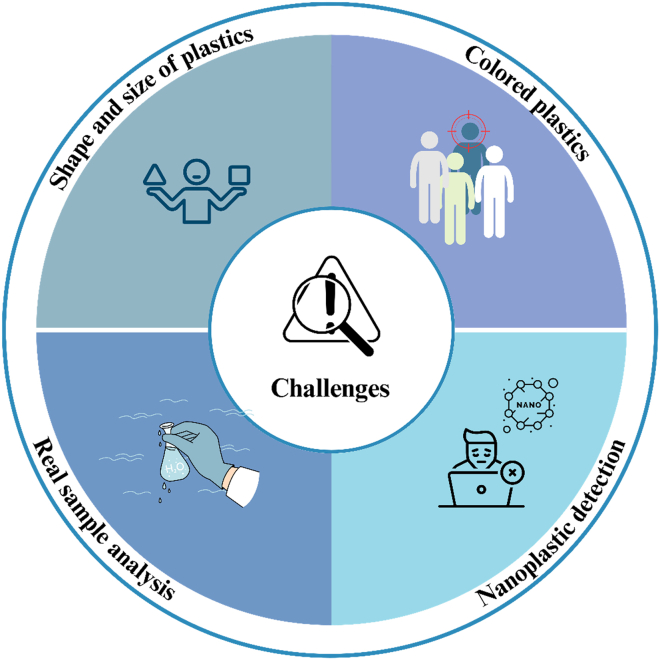
Future research developments in RS and SERS for micro/NPs detection

To address the aforementioned challenges, future efforts could focus on: (i) advancing the development of high-performance SERS substrates to improve the sensitivity and reproducibility of SERS measurements; (ii) integrating RS with complementary analytical techniques such as HPLC, mass spectrometry, and imaging methods; (iii) enhancing Raman instrumentation to achieve superior spectral, spatial, and temporal resolution; (iv) adopting emerging approaches such as tip-enhanced Raman spectroscopy (TERS)^197^^,^^198^ and shell-isolated nanoparticle-enhanced Raman spectroscopy (SHINERS).^199^^,^^200^^,^^201^ TERS couples scanning probe microscopy with localized plasmonic enhancement, enabling chemical identification with nanoscale resolution (<10 nm), reported enhancement factors of 10^6^–10^8^, and single-molecule sensitivity. SHINERS, in contrast, uses ultrathin inert shells (e.g., silica, alumina) around plasmonic cores to deliver uniform and reproducible enhancement (RSD <8%) while preventing direct chemical interference; and (v) applying AI and ML approaches to augment data interpretation and analysis.^202^ The schematic representation of the future directions in microplastic SERS is shown in Figure 9. Progress in these areas is poised to establish RS and SERS as more reliable and versatile tools for monitoring plastic pollution in food, water, soil, environmental and biological systems, and beyond.

## Conclusions

In the last few decades, the exponential rise in plastic materials usage has resulted in the widespread circulation of micro- and nanoplastics across diverse environmental and biological matrices, posing significant risks to ecosystems and human health. To address the analytical challenges with their detection and quantification, RS and, more importantly, SERS have drawn considerable attention as a powerful, non-destructive, and sensitive analytical technique for identifying these tiny pollutants at trace levels.

This review covered the fundamental principles of Raman and SERS, critically evaluated substrate fabrication strategies, and assessed their integration with advanced data analytics and ML tools. In a research study, Luo et al. applied CNN for direct analysis of six common microplastic mixtures, achieving 99.54% accuracy on raw SERS spectra, surpassing traditional methods such as SVM, principal component analysis-linear discriminant analysis (PCA-LDA), partial least squares discriminant analysis (PLS-DA), Random Forest (RF), and KNN, with or without spectral preprocessing. This demonstrates CNN as a fast and highly accurate approach for MP mixture identification without preprocessing.^203^

Raman datasets of NPs were processed with peak extraction and retention strategies, and an RF model achieved 98.8% average accuracy. The method was validated with spiked tap water (>97% accuracy) and further confirmed in rainwater, successfully detecting PS and PVC NPs. Together, these findings highlight the promise of ML-Raman strategies for the reliable monitoring of micro- and NPs in complex environments.^204^ Moreover, it underscored the growing potential of SERS in real-world sample analysis, including food, water, air, and biological samples. Although significant progress has been made, primary hurdles persist, including signal variability, lack of standardization, reproducibility, SERS substrate stability, sensitivity, and the complexity of real-sample matrices.

To unleash the full potential of Raman and SERS-based detection methods, future efforts can focus on designing reproducible, scalable, and application-specific SERS substrates, developing standardized analytical procedures for field analysis, and developing hybrid detection methods with AI-assisted data interpretation. Through such multidisciplinary efforts, Raman and SERS techniques will become indispensable tools for environmental monitoring and public health risk assessment in the war against plastic pollution.

## Acknowledgments

This work was supported by SRM University-AP through SEED funding (SRM/TRUST/AP/Jan/IC/22-23/PUR-00007). V.R. Soma acknowledges the financial support from 10.13039/501100001849DRDO, India [ERIP/ER/1501138/M/01/319/D (R&D)]. We also thank IoE, Hyderabad, for financial support through the project UOH/IOE/RC1/RC1-20-016. V.R. Soma also acknowledges the IOE, UoH, for funding through a collaborative Inter-Institutional Research Clusters Project UoH-IOE-IIRC-24-007. We thank the Director, DIA-CoE, Dr. S.C. Bhattacharya, for his encouragement and support.

## Author contributions

Writing original draft preparation and writing review and editing – J.K., writing review and editing – P.A., writing review and editing – S.F.H., visualization – R.S., conceptualization, supervision, and writing review and editing – V.R.S., conceptualization, supervision, and writing review and editing – R.P.

## Declaration of interests

The authors declare no competing interests.
